# Programming maternal and child overweight and obesity in the context of undernutrition: current evidence and key considerations for low- and middle-income countries

**DOI:** 10.1017/S1368980016003323

**Published:** 2017-01-09

**Authors:** Lindsay M Jaacks, Justine Kavle, Abigail Perry, Albertha Nyaku

**Affiliations:** 1 Department of Global Health and Population, Harvard T.H. Chan School of Public Health, Harvard University, 665 Huntington Avenue, Building 1, Room 1221, Boston, MA 02115, USA; 2 Maternal and Child Survival Program, Washington, DC, USA; 3 Maternal, Newborn, Child Health and Nutrition (MNCHN) Program, PATH, Washington, DC, USA; 4 Department of Prevention and Community Health, Milken Institute School of Public Health, George Washington University, Washington, DC, USA; 5 Department for International Development, London, UK

**Keywords:** Overweight, Dual burden, Developing countries, Programme implementation

## Abstract

The goals of the present targeted review on maternal and child overweight and obesity were to: (i) understand the current situation in low- and middle-income countries (LMIC) with regard to recent trends and context-specific risk factors; and (ii) building off this, identify entry points for leveraging existing undernutrition programmes to address overweight and obesity in LMIC. Trends reveal that overweight and obesity are a growing problem among women and children in LMIC; as in Ghana, Kenya, Niger, Sierra Leone, Tanzania and Zimbabwe, where the prevalence among urban women is approaching 50 %. Four promising entry points were identified: (i) the integration of overweight and obesity into national nutrition plans; (ii) food systems (integration of food and beverage marketing regulations into existing polices on the marketing of breast-milk substitutes and adoption of policies to promote healthy diets); (iii) education systems (integration of nutrition into school curricula with provision of high-quality foods through school feeding programmes); and (iv) health systems (counselling and social and behaviour change communication to improve maternal diet, appropriate gestational weight gain, and optimal infant and young child feeding practices). We conclude by presenting a step-by-step guide for programme officers and policy makers in LMIC with actionable objectives to address overweight and obesity.

The increasing prevalence of overweight and obesity among women and children in low- and middle-income countries (LMIC) represents hindered progress on maternal and child health, and has important implications for future economic development^(^ ^1^ ^)^. Between 2006 and 2015, the cost of lost economic productivity due to obesity-related CVD and diabetes in LMIC was estimated to be $US 84 billion^(^ ^2^ ^)^. Moreover, obesity-related disability-adjusted life years in LMIC have increased dramatically over the past two decades: from 518 per 100 000 people in 1990 to 985 per 100 000 people in 2013, which translates into a 90 % relative increase in disability-adjusted life years^(^ ^3^ ^)^. Given that one of the targets for the Sustainable Development Goal 2, ‘End Hunger’, is to end all forms of malnutrition, strategies to address both undernutrition and overweight and obesity globally are urgently needed.

The purpose of the present targeted review on maternal and child overweight and obesity was not to systematically review trends in the prevalence of maternal and child overweight and obesity in LMIC. Instead, we aimed to conduct a targeted review in order to: (i) understand the current situation in LMIC with regard to recent trends and context-specific risk factors; and (ii) building off this, identify entry points for leveraging existing undernutrition programmes to address overweight and obesity in LMIC. We focused on low-income and lower-middle-income countries, particularly in sub-Saharan Africa, where large development agencies currently support nutrition programmes. The overall goal is to spark evidence-based action in this important and almost entirely neglected topic in global nutrition.

## Trends in overweight and obesity among women and children

According to the 2016 *Global Nutrition Report*, ‘obesity and overweight are now a staggering global burden’^(^ ^4^ ^)^. A recent analysis of Demographic and Health Surveys found that in urban Mauritania, over half of reproductive-aged women are overweight or obese, and the prevalence in urban areas of Ghana, Kenya, Niger, Sierra Leone, Tanzania and Zimbabwe is approaching 50 %^(^ ^5^ ^)^. The annualized change in prevalence from approximately 2000 to 2010 among women in urban areas of Bangladesh, Ghana, Malawi, Nepal, Niger, Rwanda, Zambia and Zimbabwe was at least 1·00 %^(^ ^5^ ^)^, meaning that if trends continue, the prevalence of overweight in urban areas of these countries will increase by 10 % over the next decade. The prevalence of overweight and obesity is lower in rural areas compared with urban areas in most LMIC^(^ ^5^ ^)^. However, looking at trends over time, the recent rate of increase in overweight and obesity in several countries (e.g. Burkina Faso, Kenya, Uganda and Zimbabwe) is greater in rural areas compared with urban areas^(^ ^5^ ^)^. Thus, while rural–urban disparities persist, the gap is narrowing in some countries.

While the most recent prevalence of overweight and obesity among adolescent girls was much lower than that among women, trend data indicate that the prevalence in adolescent girls is increasing over time in many countries^(^ ^6^ ^)^. With respect to children under 5 years of age, Malawi, Mozambique, Nigeria, Rwanda and Zambia have a prevalence of overweight greater than 7 %^(^ ^7^ ^)^, which is the global nutrition target for childhood overweight set by the WHO^(^ ^8^ ^)^.

## Context-specific risk factors for overweight and obesity

Risk factors were identified via reviewing the seminal *Lancet* series on obesity (2011 and 2015) and the Bellagio Conference on Program and Policy Options for Preventing Obesity in Low- and Middle-Income Countries^(^ ^9^ ^–^ ^11^ ^)^. Shifts in dietary intake and physical activity resulting from economic development, urbanization and globalization of the food system are thought to be key drivers of the global obesity epidemic, and are described in detail elsewhere^(^ ^9^ ^,^ ^10^ ^,^ ^12^ ^)^. Several risk factors may be of particular importance in the context of vulnerable populations living in LMIC and are discussed in detail here. These include: (i) maternal nutrition and appropriate gestational weight gain; (ii) infant and young child nutrition and healthy growth trajectories; (iii) value and social standing; (iv) time and effort for food preparation; and (v) cultural beliefs about body size.

### Maternal nutrition and appropriate gestational weight gain

Few studies have explored gestational weight gain knowledge and beliefs in LMIC. One study, conducted in Nigeria, found that 40·1 and 35·4 % of mothers were classified as overweight and obese, respectively, according to pre-pregnancy BMI^(^ ^13^ ^)^. However, none of the obese mothers perceived themselves as obese^(^ ^13^ ^)^. In contrast, 32·0 % of the overweight mothers perceived themselves as overweight, and those who perceived themselves as overweight were more likely to gain the recommended amount of weight during pregnancy^(^ ^13^ ^)^. A qualitative study in Egypt found that women were not aware of and had no concept of appropriate gestational weight gain due to a lack of counselling by health-care providers^(^ ^14^ ^)^. Given that maternal obesity and excess gestational weight gain have been associated with increased adiposity in offspring, largely in studies conducted in developed countries^(^ ^15^ ^)^, this is an important research gap in LMIC. In addition, insufficient maternal intakes of energy and micronutrients have been associated with increased adiposity in offspring, particularly female offspring – as reviewed by Yang and Huffman^(^ ^16^ ^)^ and Christian and Stewart^(^ ^17^ ^)^. For example, maternal energy intake of less than 3766 kJ/d (900 kcal/d) during the Dutch famine of 1944–1945 was associated with greater fat deposition in female offspring (average age at follow-up approximately 60 years), but not males^(^ ^18^ ^)^. Two secondary analyses of trials, one in Nepal^(^ ^19^ ^)^ and one in Peru^(^ ^20^ ^)^, found increased linear growth (Nepal) and lean body mass (Peru), and, in the case of Nepal, decreased adiposity, among offspring of women who received Zn+Fe+folic acid during pregnancy *v*. offspring of women who received Fe+folic acid.

### Infant and young child nutrition and healthy growth trajectories

Fetal growth and growth in the first 2 years of life are important predictors of adult weight status^(^ ^21^ ^)^. A recent analysis of the Consortium on Health Oriented Research in Transitional Societies (COHORTS) study, which includes five LMIC (Brazil, Guatemala, India, the Philippines and South Africa), found that birth weight was more strongly associated with adult lean body mass than with adult fat mass^(^ ^22^ ^)^. Similarly, a study in Pune, India, found that the small birth size of babies was largely due to reduced skeletal muscle rather than reduced body fat^(^ ^23^ ^)^, and a study in New Delhi, India, found that birth weight was positively associated with adult lean body mass but not adult central adiposity^(^ ^24^ ^)^. In contrast, with respect to postnatal growth, a meta-analysis of fifteen studies reported an 84 % increase in risk of obesity at 10 years of age per 0·67 sd gain of weight in infancy^(^ ^25^ ^)^. Data from the COHORTS study suggest that both faster relative weight gain and faster linear growth among children were associated with increased risk of adult overweight^(^ ^26^ ^)^. However, it is important to note that the magnitude of these associations in the COHORTS study was small: a 1 sd change in weight gain up to 12 or 24 months predicted less than a 1% change in adult body composition^(^ ^22^ ^)^. Together, these results suggest two key points:

1.
improved birth weight, as an indicator of fetal growth, may be protective against overweight via building adult lean body mass; and
2.
the known benefits of growth in the first 2 years of life on health and function^(^ ^27^ ^)^ and the known adverse consequences of growth faltering in early life on adult stature^(^ ^28^ ^)^ are likely to outweigh any negative effects on adult obesity resulting from rapid ‘catch-up’ growth during this period^(^ ^26^ ^)^.

While several systematic reviews and meta-analyses, including a WHO-led review^(^ ^29^ ^)^, have found protective effects of breast-feeding on obesity in developed countries, including the USA, Canada, UK, Germany, Australia, New Zealand and former Czechoslovakia, there is a need for additional studies to provide stronger evidence because these studies did not account for publication bias and residual confounding by maternal socio-economic status and lifestyle habits^(^ ^30^ ^)^. One randomized controlled trial on promotion of exclusive breast-feeding in Belarus did not find any effect on childhood obesity^(^ ^31^ ^)^. A significant evidence gap remains on whether any relationship between breast-feeding and overweight exists in LMIC. A study in Brazil did not find a significant association between breast-feeding duration and BMI^(^ ^32^ ^)^, while a study in India found only a weak association between breast-feeding duration and BMI and no association with skinfold thickness^(^ ^33^ ^)^. Regardless of whether breast-feeding is or is not protective against overweight, the strong evidence supporting other benefits of breast-feeding^(^ ^34^ ^)^ warrants continued support of breast-feeding recommendations^(^ ^35^ ^)^.

With regard to complementary feeding, the limited data available from LMIC suggest that suboptimal complementary feeding practices are common and include energy-dense, nutrient-poor foods. For example, a survey of 700 mothers of 6- to 18-month-old infants in Ibadan, Nigeria, found that, on a daily basis, 65·0 % regularly gave biscuits to their infants, 16·1 % gave soft drinks and 9·6 % gave commercial fruit juice^(^ ^36^ ^)^. In addition, 57·0 % of mothers sweetened their infants’ *pap* (maize-based porridge) with sugar^(^ ^36^ ^)^. In a study of 4299 children living in two slums outside Nairobi, Kenya, 41 % had received sweetened/flavoured water in the first 6 months of life^(^ ^37^ ^)^. With regard to breast-milk substitutes, high-protein infant formulas in the first 2 years of life have been associated with increased adiposity in childhood relative to control formulas in several randomized controlled trials conducted in Europe^(^ ^38^ ^,^ ^39^ ^)^, but this relationship has not been explored in LMIC.

### Value and social standing

Few studies have explored drivers of food choice in LMIC. One qualitative study, conducted in rural Kerala, India, found that the two most important drivers of food choice at the household level were affordability and taste preferences of the children and husband^(^ ^40^ ^)^. For example, one woman said, ‘… we use lot of oil … difficult to reduce, he [husband] likes lot of fried things’^(^ ^40^ ^)^. Processed foods were perceived as ‘higher value’ based on the media’s portrayal of these foods as necessary for healthy growth and development of children^(^ ^40^ ^)^. This can often lead to higher-than-affordable expenditures on these foods: for example, one woman said, ‘… spend an average four-thousand rupees on food per month … have to spend an extra one to two thousand on biscuits and powder items [health drinks] for small children in the house’^(^ ^40^ ^)^. Similarly, expensive foods such as restaurant foods were considered ‘better’ than foods that were cheaper and could be prepared at home^(^ ^40^ ^)^. A study in Nigeria also reported that consumption of fast foods was associated with higher social standing^(^ ^41^ ^)^. In contrast, in Egypt, processed junk foods for children were not perceived as expensive and were within economic reach of most families in both rural and urban areas^(^ ^42^ ^)^. Also in Egypt, junk food consumption was not just an issue among children; mothers and other family members also consumed these foods^(^ ^42^ ^)^.

### Time and effort for food preparation

As women enter the workforce, their time to prepare food becomes limited. For example, in the aforementioned qualitative study conducted in rural Kerala, India, one mother stated: ‘I come back from work and I just try to make something fast for the children as they are hungry by then’^(^ ^40^ ^)^. In contrast, in lower-income households in these communities in rural Kerala, the limitation was affordability, not time: ‘Most people complain that it takes a lot of time and effort to cut vegetables. That is only for those who don’t have time. Here, we have time, but no vegetables and no money to buy it’^(^ ^40^ ^)^. In Egypt, reducing consumption of junk food is challenging because mothers often give these foods out of convenience, stating that, ‘I have no time to cook for my children’, ‘I have no free time’ and ‘I felt lazy’^(^ ^42^ ^)^. Moreover, data from Egypt suggest that older siblings care for younger children and play an important role in feeding young children when mothers are away from home^(^ ^42^ ^)^. A recent analysis of data from Cambodia found that women increasingly have sales and service jobs, especially in urban settings, and that this is linked to overweight and obesity, potentially through time pressures in addition to increased sedentary time^(^ ^43^ ^)^. Further research is needed that focuses on the impact of women entering the workforce on weight status in LMIC and on potential mechanistic pathways other than sedentary time, such as stress, time pressures and economic independence.

### Cultural beliefs about body size

Studies in some LMIC indicate that cultural beliefs about body size may be an important barrier to programmes aimed at preventing overweight and obesity. For example, in urban areas of Pakistan, where 64 % of adult study participants were overweight, most did not perceive themselves as overweight^(^ ^44^ ^,^ ^45^ ^)^. Two cross-sectional studies of overweight female university students residing in urban Pakistan reported that up to 18 % believed they were of normal weight^(^ ^45^ ^,^ ^46^ ^)^. Findings also reveal that a Pakistani adult who is happy or does not think about his/her weight is six times more likely to misperceive him/herself as not being overweight^(^ ^44^ ^)^. In some sub-Saharan African countries, such as Nigeria, obesity is perceived as a sign of ‘power, respect, and an evidence of good living’^(^ ^41^ ^)^. Further research is needed to understand beliefs relating to overweight and obesity, particularly in areas with a high prevalence of HIV/AIDS, as there may be a stigma attached to thinness in these contexts^(^ ^47^ ^)^.

## Entry points for overweight and obesity programming within existing undernutrition programmes

**Fig. 1:**
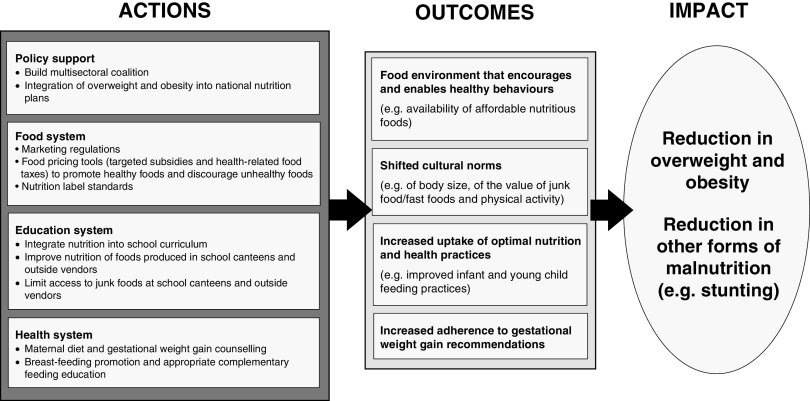
Framework illustrating how specific actions can help to achieve meaningful improvements in outcomes such as increased uptake of optimal nutrition and health practices, and how they impact overweight and obesity and other forms of malnutrition

Based on our analysis of context-specific risk factors, we developed a conceptual framework (Fig. 1) that highlights programmes and policies that could be prioritized to address overweight and obesity in LMIC. The WHO’s *Global Action Plan for the Prevention and Control of Noncommunicable Diseases 2013–2020* ^(^ ^48^ ^)^, the WHO report *Population-Based Approaches to Childhood Obesity Prevention* ^(^ ^48^ ^,^ ^49^ ^)^ and the WHO report *Interventions on Diet and Physical Activity: What Works* ^(^ ^50^ ^)^ were also consulted when developing the conceptual framework. The overlap between aspects of this framework and existing nutrition programmes largely focused on undernutrition is presented in Table 1. The four promising entry points identified were as follows:

1.
integration of overweight and obesity into national nutrition plans;
2.
food systems (integration of food and beverage marketing regulations into existing polices on the marketing of breast-milk substitutes and adoption of policies to promote healthy diets);
3.
education systems (integration of nutrition into school curricula with provision of high-quality foods through school feeding programmes); and
4.
health systems (counselling and social and behaviour change communication to improve maternal diet, appropriate gestational weight gain, and optimal infant and young child feeding practices).

**Table 1:** Examples of programmatic approaches to address overweight and obesity in low- and middle-income countries in the context of existing nutrition programmes largely focused on undernutrition

	Food systems	Education systems	Health systems
Existing nutrition programmes	∙ Restrict marketing of breast-milk substitutes ∙ Invest in food distribution infrastructure ∙ Increased access to legumes/pulses and grains high in protein and micronutrients ∙ Public procurement and distribution of fruits and vegetables	∙ Schools should develop dietary guidelines that promote and provide healthy foods and, where feasible, provide healthy breakfast and/or lunch to students	∙ Counselling by community health workers on appropriate dietary intake during pregnancy ∙ Breast-feeding promotion ∙ Appropriate complementary feeding that emphasizes quantity, quality and diversity of foods according to age, and ensures intra-household allocation of foods to infants and young children
Integration of overweight and obesity	∙ Restrict marketing of unhealthy foods to children ∙ Food-based dietary guidelines ∙ Nutrition label standards	∙ School curricula should include what constitutes a ‘healthy diet’, the health consequences of overweight/obesity and the importance of physical activity ∙ Adolescent girls are an important target population ∙ Schools should reduce access to sugary, high-fat snacks and beverages ∙ The importance of a healthy weight should also be discussed in preparation for pregnancy, with non-pregnant women and adolescents, through community channels or schools	∙ Develop overweight and obesity polices and guidelines and provide budgetary allocations for implementation at district and health facility levels ∙ Integrate overweight into pre-service and in-service training curricula of health providers ∙ Incorporate promotion of ‘healthy diets’ in nutrition behaviour change activities ∙ Heath-care providers should provide counselling on maternal diet and appropriate gestational weight gain during antenatal care ∙ Counselling should be based on an understanding of cultural beliefs and food choice related to high-energy foods (i.e. sugary foods and fried foods) and nutritious foods (i.e. fruits and vegetables) ∙ Counsel mothers, caregivers, family members and influential community members to feed children appropriately and to not consume junk foods ∙ Avoid providing sugar-sweetened beverages during community/mothers’ support group sessions and postnatal care visits ∙ Develop monitoring and evaluation indicators and targets for national, district/health facility and community levels ∙ Include overweight indicators in national development plans

### Integration of overweight and obesity into national nutrition plans

Strong nutrition governance, including setting SMART (specific, measureable, achievable, relevant and time-bound) targets, has been linked to achieving undernutrition goals such as stunting^(^ ^4^ ^,^ ^51^ ^)^. Few LMIC address overweight and obesity in their national nutrition plans. Kenya is an example of one such country. The Kenyan National Nutrition Action Plan (2012–2017) outlines specific activities to address the increase in overweight and obesity in Kenya, including the following: review, develop and disseminate a comprehensive strategy and guidelines for preventing, managing and controlling nutrition-related non-communicable diseases; train service providers and create public awareness on the importance of preventing, managing and controlling nutrition-related non-communicable diseases; scale up community screening of BMI and waist circumference; and improve nutrition in schools. Kenya’s 2013 National Maternal, Infant, and Young Child Nutrition Policy Guidelines also state that childhood obesity is an emerging public health problem.

### Food systems

The food system is a critical underlying determinant of dietary intake, and includes food production, distribution, processing, packaging, marketing and retail. Two aspects of food systems that are particularly important for addressing overweight and obesity in LMIC include: (i) implementing the WHO recommendations on marketing of food and beverages to children; and (ii) policies that promote healthy diets and discourage unhealthy diets.

According to the WHO, introducing policies that reduce the marketing of foods high in saturated fat, *trans*-fat, free sugars or salt to children is likely to be one of the most cost-effective interventions available to governments to address obesity^(^ ^49^ ^,^ ^52^ ^)^. Three upper-middle-income countries (Brazil, Thailand and South Africa) have drafted resolutions that address food marketing to children^(^ ^53^ ^)^, but these policies are currently suspended in both Brazil^(^ ^54^ ^)^ and South Africa^(^ ^55^ ^)^. Many LMIC have already adopted policies that regulate food industry marketing of breast-milk substitutes^(^ ^56^ ^)^ and lessons learned from that process may prove to be informative for adopting resolutions to regulate marketing of unhealthy foods to children^(^ ^57^ ^)^. Lessons learned from high-income countries that have already adopted marketing restrictions could also be useful to policy makers in LMIC. For example, in the UK, where marketing restrictions have been in place since 2007, an analysis of advertising exposure to high-fat, -salt or -sugar products among children under 16 years of age found almost universal adherence to the restrictions, but that exposure did not change before and after the adoption of the restrictions^(^ ^58^ ^)^, suggesting that stronger restrictions targeting a wider range of advertisements is needed.

The NOURISHING framework developed by World Cancer Research Fund International is a tool that policy makers in LMIC can use for guidance on developing a comprehensive policy package to promote healthy diets and discourage unhealthy diets^(^ ^59^ ^)^. Few LMIC have adopted policies consistent with the NOURISHING framework. This was made evident at the 2013 Bellagio Conference on Program and Policy Options for Preventing Obesity in Low- and Middle-Income Countries^(^ ^9^ ^)^: of the thirteen countries represented at the Conference, only two were lower-middle-income countries (India and Bangladesh) and none were low-income countries. Nevertheless, some LMIC policies addressing the food system within the NOURISHING framework are worth mentioning here.

Several LMIC have policies that promote fruit and vegetable production; many were originally adopted in order to address micronutrient deficiencies, but now play the additional role of overweight and obesity prevention^(^ ^60^ ^)^. Several LMIC have also adopted food-based dietary guidelines. For example, the Dietary Guidelines for the Brazilian Population, which, in conjunction with the National Food and Nutrition Security Policy and National Health Promotion Policy, aim to promote healthy eating and the prevention of malnutrition including micronutrient deficiencies and overweight and obesity^(^ ^61^ ^)^. These Guidelines could be adapted to other countries as part of a comprehensive policy package to address malnutrition, including overweight and obesity.

In 2005, the Ghana Ministry of Health adopted the Regenerative Health and Nutrition Program with the primary objective of promoting healthy lifestyles, including diet and daily physical activity. Since 2006, over 50 000 community members have been trained, who in turn educate other community members. However, analysis of data from before and after implementing the programme shows an overall decline in unhealthy behaviours only among highly educated individuals^(^ ^62^ ^,^ ^63^ ^)^. Moreover, a panel in the 2016 *Global Nutrition Report* suggests that the 2012 National Policy for the Prevention and Control of Chronic Non-Communicable Diseases in Ghana has not been ‘operationalized in any way’^(^ ^4^ ^)^.

Finally, nutrition labelling has been recommended as a policy tool for addressing unhealthy food environments^(^ ^49^ ^)^. However, the impact of interpretive nutrition labelling policies on food purchasing patterns has been evaluated only in the UK, where there was ‘no discernible effect on the relative healthiness of consumer purchases’^(^ ^64^ ^)^.

### Education systems

A systematic review including twenty-two studies from LMIC found that school-based interventions have the potential to improve diet and physical activity behaviours and decrease BMI in these contexts^(^ ^65^ ^)^. Schools offer a unique opportunity to change norms around nutritional practices and healthy body weights. The review also found that involving multiple stakeholders and integrating educational activities into existing school curricula worked best^(^ ^65^ ^)^.

Two cities in Africa – Ouagadougou, Burkina Faso and Cotonou, Benin – have piloted the WHO Nutrition-Friendly School Initiative, which is a framework for the prevention of the double burden of undernutrition and overweight among schoolchildren. There are five conditions that must be met for a school to be considered nutrition-friendly, beginning with the formation of a School Nutrition Committee that involves key stakeholders. The School Nutrition Committee is responsible for meeting these five conditions and for monitoring and evaluation of the programme: (i) a written school policy on nutrition; (ii) building awareness and capacity of the surrounding community as they relate to nutrition; (iii) school curriculum adaptation to include nutrition; (iv) school environment that supports optimal nutrition and health; and (v) school nutrition and health services^(^ ^66^ ^)^. In Ouagadougou, sensitizing communities to nutrition was identified as a priority for half of the schools^(^ ^67^ ^)^, and many involved in the initiative felt that these policies should be implemented at the national level rather than the school level^(^ ^67^ ^)^. Piloting the Nutrition-Friendly School Initiative in 2007 in New Delhi, India, an initial assessment found no written nutrition policies in the four participating schools and no integration of nutrition and health into the school curriculum^(^ ^68^ ^)^.

A 6-month nutrition education programme focused on nutrition, physical activity, non-communicable diseases and healthy cooking practices was implemented in both private and public schools of three northern Indian cities (New Delhi, Agra and Jaipur)^(^ ^69^ ^)^. At baseline, only 25–55 % of students considered deep-fried Indian snack foods to be junk foods and 25–35 % of students thought that consuming butter improves bone strength and health^(^ ^69^ ^)^. Results of the intervention were promising: a pre/post statistical comparison of responses of a random subset of students to a questionnaire on health and nutrition-related knowledge found significant improvements in both private and public schools, particularly on questions relating to physical activity and healthy cooking practices^(^ ^69^ ^)^.

Government policies to support the provision of healthy foods in schools could build off the Purchase from Africans for Africa programme (Ethiopia, Malawi, Mozambique, Niger and Senegal), which encourages the consumption of non-processed, nutrient-dense foods (cereals, pulses and legumes, fruits, vegetables and animal products)^(^ ^70^ ^)^. For example, as part of the programme, Malawi smallholder farmers now provide staple grains, groundnuts, bananas and fish to ten primary schools where enrolment has increased by 15 %^(^ ^71^ ^)^. Prevention of overweight and obesity could be an important co-benefit of such programmes.

### Health systems

Currently, programming during routine contact points with women and children at the community and facility levels in LMIC focuses on reducing undernutrition without consideration of overweight and obesity. However, these are unique opportunities to address maternal diet and weight gain during pregnancy, as well as infant and young child feeding practices that are important risk factors for overweight and obesity.

Few studies in LMIC have evaluated the impact of antenatal counselling on appropriate gestational weight gain. In Nigeria, receiving information on gestational weight gain from multiple health-care providers was significantly associated with increased levels of maternal knowledge on risks of inappropriate weight gain^(^ ^13^ ^)^. However, although 57 % of Nigerian mothers were counselled on the risks associated with excess weight gain, many mothers were not aware of the recommended amount of weight gain during pregnancy^(^ ^13^ ^)^. In Egypt, women were also not counselled on weight gain during pregnancy and had little understanding of the appropriate amount of weight gain^(^ ^14^ ^)^. A small study of women attending a clinic in Accra, Ghana, found that they were willing to reduce their body size in order to reduce the risk of obesity-related illnesses such as hypertension, diabetes, CVD and myocardial infarction^(^ ^72^ ^)^. Education, income, marital status and number of children did not influence their response, although women who were younger than 50 years of age tended to be more likely to report being willing to reduce their body size to improve health^(^ ^72^ ^)^. Thus, providing counselling on maternal diet and weight gain during pregnancy during antenatal care can potentially address overweight and obesity in LMIC.

**Fig. 2:**
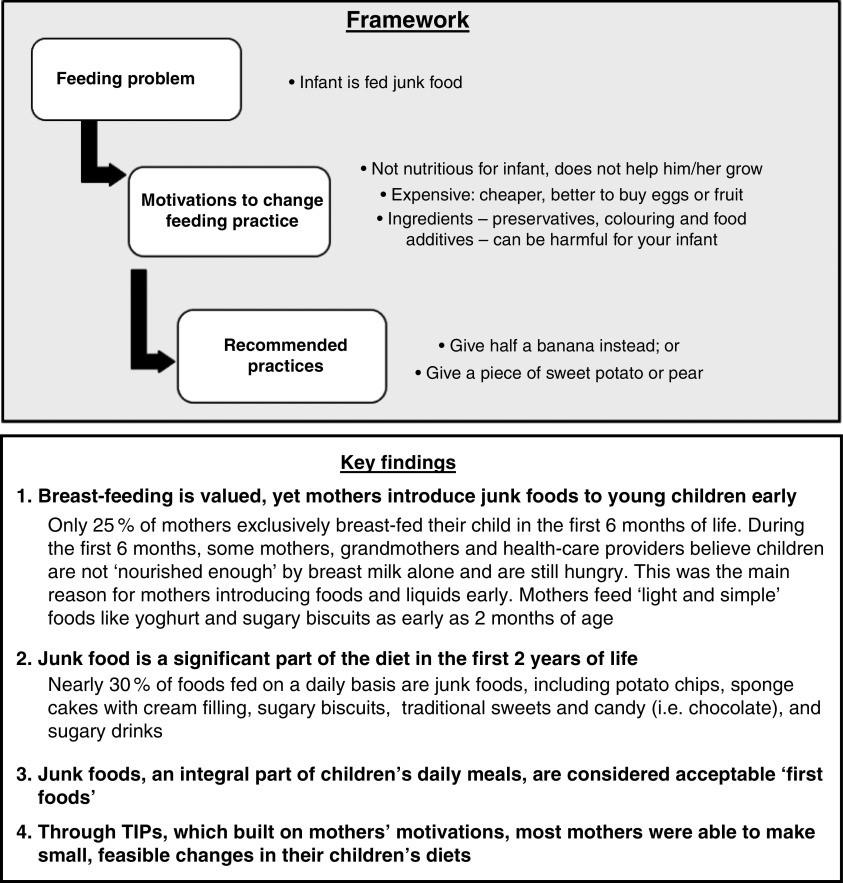
Example of how Trials for Improved Practices (TIPs) addressed junk food as an infant feeding problem in Egypt

Another important point of entry is existing health promotion-based interventions such as complementary feeding interventions for infants and young children. Health-care providers and families need to understand healthy *v*. unhealthy weight gain and can learn to monitor excessive and/or rapid weight gain within the context of undernutrition programming. In addition, families should be advised that junk foods are detrimental to the growth of children and the entire family’s health, and providers should be trained to provide nutrition counselling for families during routine growth monitoring visits^(^ ^73^ ^)^. A recent study in Egypt used Trials for Improved Practices (TIPs) methodology to address poor infant and young child feeding practices over the course of three home visits (Fig. 2)^(^ ^42^ ^)^. During the first visit, researchers discussed behaviours, practices and motivations for infant and young child feeding with mothers, focusing on junk foods. During the second visit, mothers jointly decided with the research team what specific feeding practices they would be willing to try over a one-week period. During the third and final visit, mothers discussed their experiences with TIPs: which recommended practices worked, which did not work and any modifications that were made. Results supported the importance of reducing junk food consumption keeping cultural influences and beliefs in mind when designing interventions.

## Research gaps

Important research gaps identified in the present review include:

∙ Underlying drivers that are shared by all forms of malnutrition.
∙ Evaluation of the appropriateness of the Institute of Medicine guidelines for weight gain during pregnancy^(^ ^74^ ^)^ in the context of LMIC.
∙ Studies on the association between maternal overweight and obesity, gestational weight gain and child health outcomes.
∙ Studies of the long-term impact of dietary intake during infancy and early childhood on adult overweight and obesity.
∙ Drivers of food choice, especially among overweight mothers, and how this information can be used to design programmes to engage women and their families.
∙ The impact of women entering the workforce on weight status in LMIC and potential mechanistic pathways other than sedentary time such as stress, time pressures and economic independence.
∙ In settings where undernutrition is still prevalent, testing the effectiveness of interventions that integrate infant/child overweight prevention into antenatal/postnatal care.
∙ Studies of the potential adverse effects of supplemental feeding programmes on overweight. One analysis of infant feeding programmes in Chile found increases in child weight-for-age and weight-for-length with little impact on length-for-age^(^ ^75^ ^)^. A secondary analysis of a randomized controlled trial in Burkina Faso found that women with pre-pregnancy BMI in the highest tertile (21·8–28·1 kg/m^2^) who received lipid-based nutrient supplements gave birth to infants with higher leptin concentrations compared with women with pre-pregnancy BMI in the lowest tertile (15·8–19·7 kg/m^2^), which may be a marker of higher neonatal fat mass^(^ ^76^ ^)^. Thus, supplemental feeding programmes should be targeted to high-risk beneficiaries for energy supplementation (e.g. in humanitarian emergencies and to severely undernourished individuals).

## Next steps and conclusion

Outside extreme conditions, such as war and famine, no country has witnessed a decline in the prevalence of adult obesity^(^ ^11^ ^)^. Current expert opinion holds that a package of initiatives including multisectoral policies and behaviour change interventions is needed^(^ ^11^ ^)^. However, the current review has identified many substantial research gaps; this topic remains a severely neglected area of global nutrition. Thus, a key limitation of the review was the fact that there was not enough scientific evidence to support a systematic review and meta-analysis of specific programmes and policies to address overweight and obesity in LMIC. In addition, to date, programmes have struggled with operationalizing overweight and obesity within the context of undernutrition, and programmatic and routine data from health systems are largely non-existent. The goal of the present targeted review is to guide research and the design and implementation of nutrition programming in the coming years to inform such a systematic review.

**Fig. 3:**
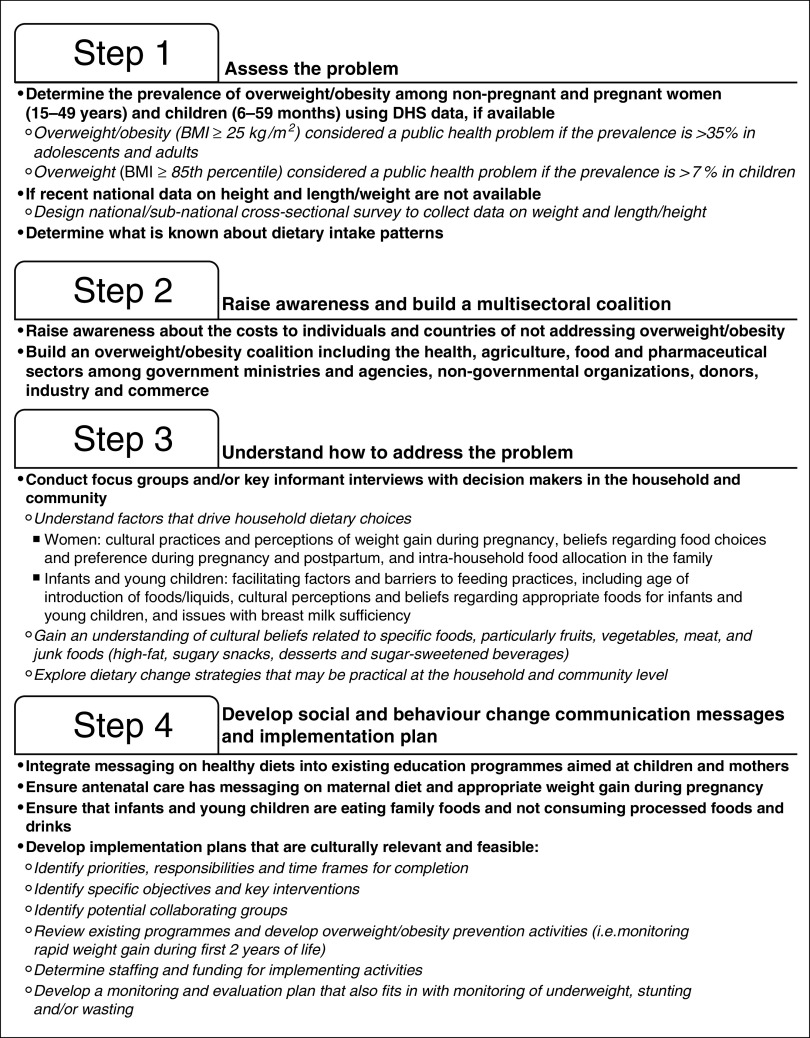
Step-by-step implementation guide to address overweight and obesity in low- and middle-income countries (DHS, Demographic and Health Survey)

The first step to addressing overweight and obesity in LMIC is integration into national nutrition plans and setting SMART targets. Nevertheless, even with these commitments, implementation of overweight and obesity prevention programmes and policies will be a challenge. To aid in this process, we have developed a four-step implementation guide for programme officers and policy makers in LMIC (Fig. 3): (i) assess the problem; (ii) raise awareness and build a multisectoral coalition; (iii) understand how to address the problem; and (iv) develop social and behaviour change communication messages and an implementation plan.

The 2016 *Global Nutrition Report* concluded that in low-income countries, the traditional issues of wasting, stunting and micronutrient deficiencies persist in the face of rising overweight and obesity^(^ ^4^ ^)^. Integration of efforts to address overweight and obesity within existing programmes focused on undernutrition is needed to maintain gains in maternal and child health in LMIC^(^ ^1^ ^)^. The food system, education system and health system could all be leveraged in the development of comprehensive programmes and policies to address all forms of malnutrition.
